# THROUGH THE LOOKING GLASS: Real-Time Imaging in Brachypodium Roots and Osmotic Stress Analysis

**DOI:** 10.3390/plants8010014

**Published:** 2019-01-08

**Authors:** Zaeema Khan, Hande Karamahmutoğlu, Meltem Elitaş, Meral Yüce, Hikmet Budak

**Affiliations:** 1 Molecular Biology, Genetics and Bioengineering Program, Faculty of Engineering and Natural Sciences, Sabanci University, Istanbul 34956, Turkey; zaeemakhan@sabanciuniv.edu; 2 Mechatronics Program, Faculty of Engineering and Natural Sciences, Sabanci University, Istanbul 34956, Turkey; khande@sabanciuniv.edu (H.K.); melitas@sabanciuniv.edu (M.E.); 3 Sabanci University SUNUM Nanotechnology Research and Application Centre, Istanbul 34956, Turkey; meralyuce@sabanciuniv.edu; 4 Cereal Genomics Lab, Department of Plant Sciences and Plant Pathology, Montana State University, Bozeman, MT 59717, USA

**Keywords:** Brachypodium, neutral red, root, Casparian bands, PEG-6000, osmotic stress, real-time imaging, PDMS

## Abstract

To elucidate dynamic developmental processes in plants, live tissues and organs must be visualised frequently and for extended periods. The development of roots is studied at a cellular resolution not only to comprehend the basic processes fundamental to maintenance and pattern formation but also study stress tolerance adaptation in plants. Despite technological advancements, maintaining continuous access to samples and simultaneously preserving their morphological structures and physiological conditions without causing damage presents hindrances in the measurement, visualisation and analyses of growing organs including plant roots. We propose a preliminary system which integrates the optical real-time visualisation through light microscopy with a liquid culture which enables us to image at the tissue and cellular level horizontally growing Brachypodium roots every few minutes and up to 24 h. We describe a simple setup which can be used to track the growth of the root as it grows including the root tip growth and osmotic stress dynamics. We demonstrate the system’s capability to scale down the PEG-mediated osmotic stress analysis and collected data on gene expression under osmotic stress.

## 1. Introduction

Abiotic stress-related research in plants has considerably increased in recent years as a result of the constant change in the global climate conditions [1,2]. Plant growth under stress conditions is generally phenotyped and visualized by macroscale parameters [3], which requires dedicated greenhouse space, labour, a great deal of test sample and consumables, thus, limits the number of parallel experiments. Also, conventional plant growth techniques are not always compatible with state-of-the-art characterization tools, such imaging tools due to the optical transparency issues of the soil pots preventing microscopic analyses at high-resolution, and the out of plane growth on agar plates hindering imaging on a single plane of focus. Engagement of microfabricated fluidic systems with plant biology research has paved the way for large-scale plant growth studies and precise morphological and physiological analyses at microscale with reduced cost and labour [4]. Those pioneering devices have allowed miniaturisation of the individual experiments and related costs while providing automated parallel assays to achieve accurate as well as high-throughput quantitative data [4,5]. Arabidopsis thaliana [6,7,8,9,10,11,12,13], Camellia Japonica [14,15,16,17], Oryza sativa [18], Nicotiana tabacum [19,20], Phalaenopsis Chiada Pioneer [21], and Physcomitrella patens [22] were the plants that have been employed in various microfluidic platforms for in-depth optical analysis of the dicot seed germination, leaf development, cell phenotypes, protoplasts, pollen tube development and dynamics, shoot and the root growth. As summarized in Table 1, these studies have primarily been carried out in dicot plants and studies on monocot plants are still to be explored.

Roots are responsible for water and mineral nutrient uptake from the soil. They offer structural stability to the plant and affect the growth and development of the plant organs above the soil. Characterization of the root behaviour at different developmental stages and under various environmental conditions is of great importance to reveal the plant tolerance mechanisms and dynamic changes, especially the ones taking place in the root systems of important food cereal crops such as wheat, rice, maize, and barley. However, conventional techniques for root investigations are usually conducted at the macro scale and do require relocation of the plants for microscopic analyses, causing dehydration or damage, thus, the data shortage. Also, the conventional tools do not allow real-time observation of the changes in the root systems that are exposed to different stress conditions such as drought, salt, growth factors, drug or nanomaterials.

To analyze temporal changes occurring under osmotic pressure and to maintain continuous imaging data during the stress application in developing seedlings, the long-term analysis in a proper setup is a requisite. To allow such imaging, the specimen must be under continuous nutrient supply while it is simultaneously accessible for visualization. While several studies of young Brachypodium roots have been carried out [23,24] the real-time optical imaging of developing roots under stress has not been reported so far, due to the technicalities mentioned above. Polydimethylsiloxane devices offer several advantages to create platforms for manipulating thick tissues to subsequently allow live imaging of cellular dynamics. These platforms can be created into desirable designs, size and tailor-made structures for the sample under observation to allow high throughput imaging data. These PDMS chips can be made into high-resolution microfluidic chips to visualize cellular dynamics or molded into larger organ-on-a-chip for tissue and plant-on-a-chip for whole plant growth analysis [12,22]. To capture long-term adaptations of plant roots to different microenvironments several root chip microfluidic platforms have also been introduced.

Recently, several chip platforms have been reported for tip-growing cells, such as in root and pollen tube growth, microenvironment investigation mostly in ornamental dicotyledonous plants [14,17]. Although all these systems attempt to mimic the physical microenvironment and provide appropriate designs for analysing spherical seeds or pollen tube elongation, there exists a need for a platform capable of measuring the elongation and growth dynamics of a model monocot plant which differs considerably in its seed architecture and germination behaviour at the cellular level. The application of abiotic stress conditions at the microscale to the monocot seeds may allow phenotyping of the most important staple food crops (having elliptical slender long grains) and be a valuable resource for a better understanding of the crop adaptation with high precision. While the analyses of several plant organs, organogenesis, pattern formation, growth dynamics have been observed in microenvironments, the structures observed have been at the cellular level with few dicotyledonous species observed Table 1. There has been little manipulation of monocot grain seedlings in such systems partly due to the size, scale and morphological intricacies of most grasses. Monocotyledonous seeds are usually elliptical, long slender grains, with embryo polarity which makes the germination behavior at the tissue and cellular level distinct from dicotyledons.

Here we report the growth of monocot seeds from model plant Brachypodium distachyon [3,25,26,27,28,29] in a polydimethylsiloxane (PDMS) based microfluidic channel in which the effects of abiotic stress on the root development were investigated in real time with various microscopy studies. The Brachypodium root has emerged as a feasible model system to study cereals organogenesis since grain grasses have either huge roots, e.g., maize, multiple roots, e.g., wheat or specialized water conditions, e.g., rice [30]. It offers various advantages for live imaging such as its simple architecture with a single primary axile root until 3 leaf stage, its moderate transparency, small size and steady growth rate [24,31]. The microfluidic channel system allowed the positioning of monocot Brachypodium seeds at the serially arranged microchannels where the root-cell microenvironment can be precisely controlled, watered, visualised in real-time, and desired stress conditions can be established. Earlier microscopic studies have been done on the morphology [32,33], growth [34] and development [35] of Brachypodium and our study focuses on the real-time growth dynamics and drought conditions in young seedlings.

## 2. Results

### 2.1. Plant Growth in PDMS

PDMS strips with single, double and triple punches were tested for compatibility with Brachypodium seedling growth, presented in Figure S1. Single, double and triple punch channels had volume capacities of 130, 280 and 385 µL, respectively. The growth compatibility and directionality observed in Figure S1a–c provided initial data on how to handle the seed polarity in wells. Growth in the device was observed until the 3-leaf stage shown in (Figure S1d,e) which showed the plausibility of growing a grain seed relatively larger than Arabidopsis in a minimal quantity of growth media. In the 3-punch preliminary device with the 385 µL MS media capacity, five weeks of growth inside the Petri plate was achieved by refilling the wells with unsolidified agar every week. Growth was observed until the formation of a small adult plant (6 leaf stage) and this observation was comparable to the plant-on-a-chip setup, reported previously for Arabidopsis [11].

**Figure 1:**
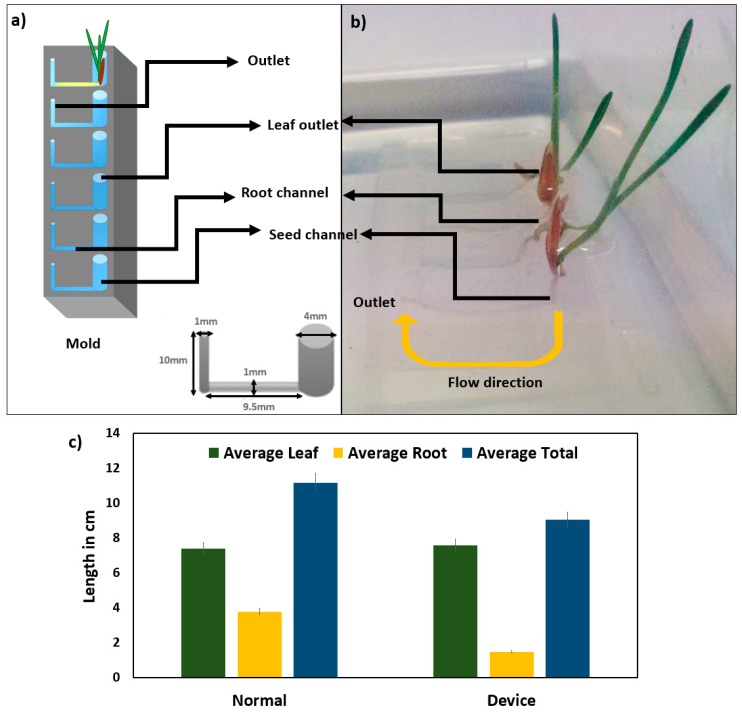
PDMS mold for growth and visualisation analysis. The mold used to construct the PDMS plant chip device (**a**,**b**) and comparison of the leaf and root growth in solid MS media plates and the plant chip device (**c**). Standard deviations were calculated from the mean values of the three samples.

Based on the observed fundamental growth dynamics, a microfluidic device was constructed using a 3D printed mold that was presented in Figure 1a,b. The monocot seedling growth in this device in solid and liquid media after vernalization and synchronous growth was comparable to the growth in horizontal agar plates as presented in Figure 1c. Two to the three-leaf stage of the seedlings on plant microfluidic chip (on the 13th day) showed a standard growth trend in the device channels filled with 385 µL of MS media. The growth rate and pattern were identical to the normal healthy development of plants in 20 mL MS agar media. The growth of the monocot seedlings in the microfluidic device

### 2.2. Microscopy Setups

**Figure 2:**
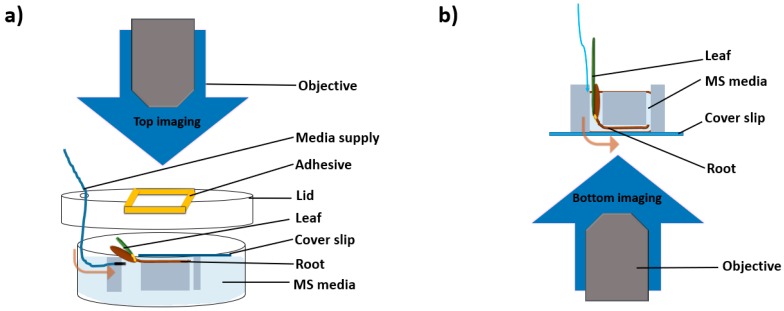

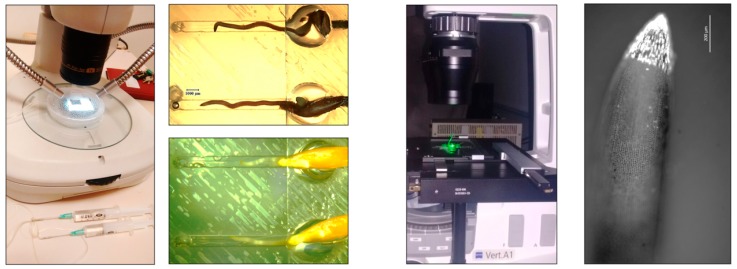
Two experimental setups for imaging were illustrated. Top imaging (**a**) and bottom imaging (**b**). Three days-old seedlings having roots were mounted into wells for the top and bottom imaging. Top imaging studies were conducted with a Nikon SMZ 1500 stereomicroscope (Japan) while the fluorescent imaging studies were conducted with a Zeiss Axio Vert.A1 inverted microscope (Germany). The mold dimensions were 10 mm height, 9.5 mm channel length, 1 mm outlet diameter, and each seed channel 4 mm in diameter.

For visualisation of the growth, the model PDMS device was used in both dorsal and ventral positions. The top imaging was achieved by plasma bonding the glass to the dorsal side, but only covering the root channel and the outlet channel, leaving the seed channel open for insertion, as can be seen in Figure 2a. Petri plate was used for maintaining humidity and growth in which the radicula was inserted into the channel, with the coleoptile facing upwards and outwards the gap created in the lid to ensure growth for the shoot. The coverslip was attached to the lid with a strong adhesive. The objective was positioned in the gap to focus directly on the cover glass. Two holes were bored inside the lid for inserting the valves for constant media flow. This entire setup was prepared aseptically under laminar flow hood. However, the seed part for shoot growth was kept uncovered. Media was inserted into the dish, and the device wells with the metalheads bored into the seed channel to ensure full media flow. The Petri plate lid and the bottom part was then covered with paraffin film to ensure high humidity. The device could be maintained in this manner for 48 h. In Figure 2b as mentioned before due to the size of the monocot seed more than two parallel experiments could not be observed simultaneously. However, the synchronous growth of 2 channels was analysed in the top setting.

The fluorescent bottom imaging was achieved by oxygen plasma binding the device to the entire ventral of the device, and the seeds were inserted into the device with the coleoptile and radicula facing outwards. The objective directly visualised the roots. Figure 2b shows the direct focus of the fluorescent microscope on the cover glass with 0.17 mm thickness to enable fluorescence. The root was separately stained with a Neutral Red dye according to the protocol by Dubrovsky et al. [43] and then rinsed in MS media and the channels filled with non-stained MS media to avoid background. For fluorescent imaging 0.4 uM, the neutral red solution was enough for the Brachypodium, and a 15–20 min incubation stained the cells sufficiently. 20% PEG was applied to full strength MS media for the stress analyses under a microscope. Brachypodium seedlings were taken 48 h after the germination and stress induction by PEG %20 was shown through 18 h. The bottom imaging setting allowed the imaging of a single channel at a time but provided an accurate fluorescent signal for comparison of stress and control samples.

### 2.3. Time Lapse Growth Curve Analysis

**Figure 3:**
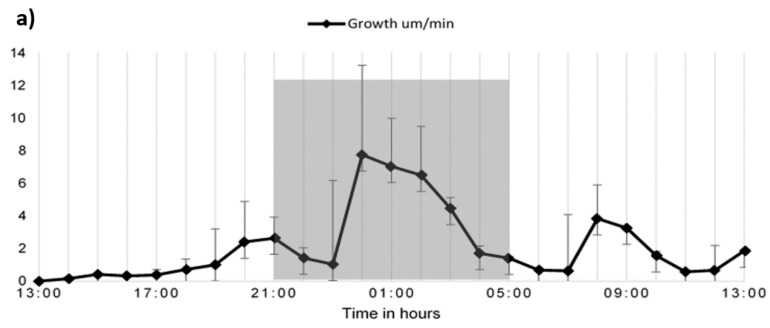

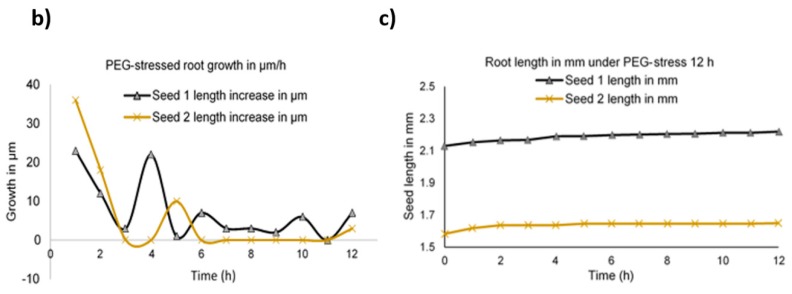
Growth trends of Brachypodium under normal and osmotic stress. The growth curve of the seedlings in 24 h (**a**). The growth trend of two seedlings under PEG stress for 12 h (**b**,**c**). The coloured lines depict two seedling roots. B is the growth in mm per minute, and C shows the overall change in length in the root over time. Standard deviations were calculated from the mean values of three independent seedlings.

The growth rate of three independent monocot seedlings in the plant chip device under 16 h day and 8 h night conditions was observed with top imaging using the Nikon microscope, Figure 3a. The 24 h time-lapse video of Brachypodium seedlings was recorded at 24 °C with a relative humidity of 37.5%. night hours. This observation could be due to negative phototropism in roots. It was observed as a general trend that plant roots grow more rapidly in the dark hours as compared to the light hours as seen in the graph from time point 20.00–4.00. A slight surge in root growth was observed in the early morning hours followed again by a steady and continuous decrease.

With time lapse recording, per minute and per hour growth was recorded and the growth over 24 h was also monitored. In the plant chip device, the growth per minute was 4.3 µm min^−1^. The growth over 24 h in Brachypodium showed a similar trend to that seen in Arabidopsis in previous studies [8,44]. In the same setup 20% PEG-mediated osmotic stress was given, and the growth of the roots for 12 h was observed (Figure 3b,c). A drop in the growth of roots was observed with almost continuous cessation of growth after 2–4 h of osmotic stress as be downfall trend in growth in µm in Figure 3b, and a constant root length in C. The length of both seedlings showed overall no growth under the osmotic stress. Since the growth of the root showed a plateau after approximately 4 h, we choose 6 h as the definite starting point to observe stress under fluorescence for morphological changes in the root.

### 2.4. Abiotic Stress Analysis by Microscopy

**Figure 4:**
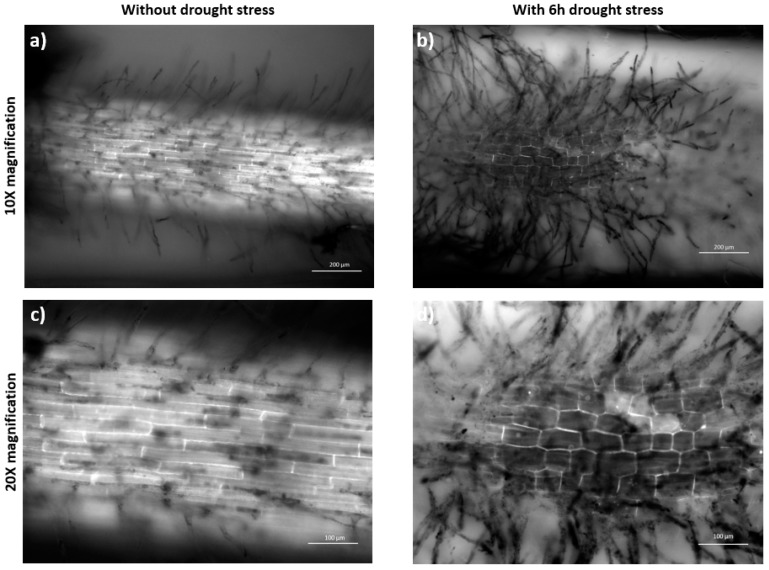
Fluorescent microscopic comparison between stressed and standard sample. Growth in the microfluidic device after 72 h, maturation zone cells under normal conditions (**a**,**c**) and after 6 h osmotic stress by 20% PEG (**b**,**d**). The images were taken with an Axio Vert.A1 inverted microscope by Carl Zeiss (Germany).

Figure 4 shows the maturation (differentiation) zone that turned to be square-like large compartments following 6 h osmotic stress by PEG 20% in comparison to the longitudinal cells observed during the normal growth. Also, the growth of several lateral roots was observed in the stressed samples, indicating an adaptive behaviour of the cells to expand the space and surface area for further water uptake [45].

**Figure 5:**
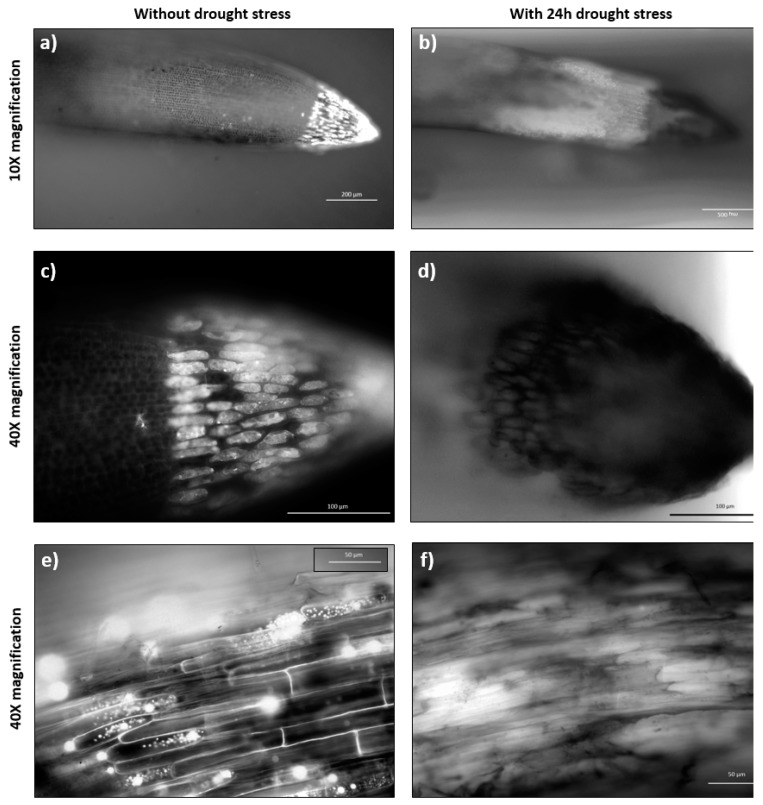
Root tip and maturation zone under osmotic stress. Root in the microfluidic device after 72 h growth, under standard and 24 h stress conditions by 20% PEG; (**a**,**b**) show the root cap samples with 10× magnification; (**c**,**d**) show the root cap samples with 40× magnification; (**e**,**f**) shows the maturation zone with 40× magnification. The images were taken with an Axio Vert.A1 inverted microscope by Carl Zeiss (Germany).

A study on young wheat seedlings also confirms such cell wall expansion in the maturation zone upon a low water potential around the roots and the authors suggest the accumulation of some solutes within the elongation and maturation zones in order to maintain the turgor pressure, resulting in an increase in the root diameter [46]. Although not the maturation zone cells, the similar swelling behaviour of the cells at the root apical meristem zone upon treatment with 5% PEG was previously reported for Brachypodium as well as wheat, rice, soybean, and maize [47], suggesting a collective response by the root tissues of different plants to surmount the osmotic stress. In accordance with these results, Figure 4 shows the cross-section images of the maturation zone cells obtained from the plants under standard (Figure 4a,b) and 24 h osmotic stress conditions (Figure 4c,d), which indicates the abnormal differentiation within the stele region of the sample under 24 h osmotic stress induced by PEG. On the other hand, the metabolic activity demonstrated by the organelles appears to be higher in the root cap cells under the standard growth conditions. However, no fluorescence was observed in the external root cap cells, which are the first sites of the plant in direct contact with the osmotic stress induced by the PEG molecules, as can be seen in Figure 5a–f.

**Figure 6:**
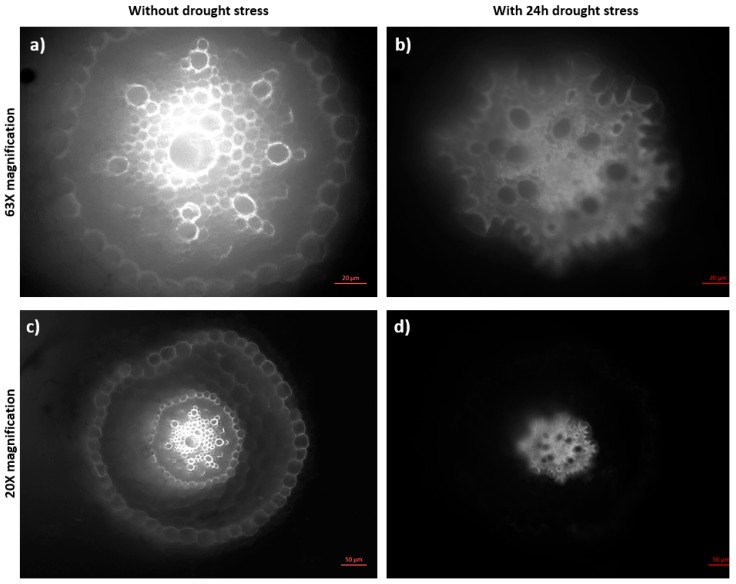
Cross section of root zones under normal and stress conditions. Root was grown in the microfluidic device for 72 h, under standard and 24 h stress conditions by 20% PEG; Cross sections were taken near root cap (**a**,**b**) and apical meristem zones (**c**,**d**) and visualized by Axio Vert.A1 inverted microscope by Carl Zeiss (Germany).

**Figure 7:**
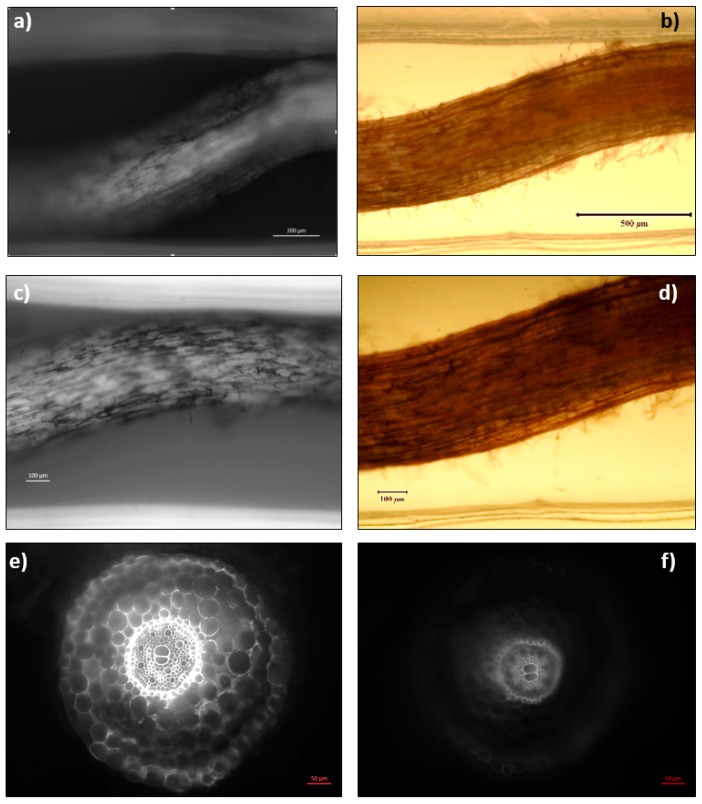
Neutral red staining of root seen under light and fluorescent microscopy. Neutral Red staining of the root with (**a**,**c**) and without (**b**,**d**) fluorescence visualization was seen after 24 h, osmotic stress mostly concentrated in the internal vascular tissue. However, a reduction in the fluorescence was observed after 24 h stress in all samples. Cross-section images of elongation zone from a control sample (**e**) and the sample under 24 h osmotic stress (**f**). Elongation zone sample was sectioned 7 mm away from the root tip. The images “a, c, e and f” were taken with an Axio Vert.A1 inverted microscope by Carl Zeiss (Germany), and the images “b” and “d” were taken with the Nikon stereomicroscope (Japan).

In Figure 6, cross section images taken 1.5 mm (around the tip) and 3 mm beneath (around the apical meristem) the root tips of the standard (Figure 6a,c) and the stressed samples (Figure 6b,d) additionally confirmed the reducing fluorescent signals as well as deformation of the cells in the sample under 24 h osmotic stress, as presented. The morphology of the midsection of the root was also analysed with and without fluorescence as seen in Figure 7a–d. The striations of live and dead cells can be differentiated by the fluorescence of neutral red, which looks concentrated around the vascular cylinder rather than the peripheral cells. The staining appeared intense within the internal cells around the stele zone and not on the peripheries which were in direct contact with PEG, indicating a hindered growth which was confirmed by fluorescence microscopy after 24 h. Cross-section images of the elongation zones from standard and stressed plant samples also confirmed the decreased fluorescent signals around the peripheries of the plant under 24 h osmotic stress, as shown in Figure 7e,f. Besides, the osmotic-stressed plants (3 and 4 days old after 24 h osmotic stress) showed re-growth on MS media plates and next planted into the pots. This observation of Brachypodium roots under osmotic stress and after the stress at early seedling stage (less than a week old) suggested remarkable tolerance of Brachypodium to high osmotic stress even at a fragile early growth stage.

### 2.5. Gene Expression Analysis

**Figure 8:**
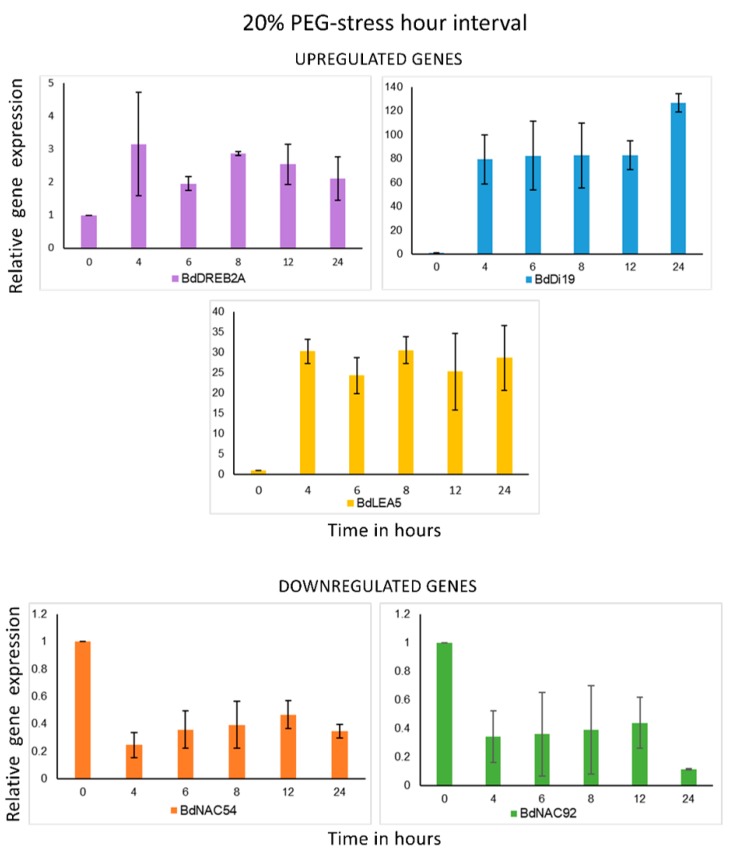
Gene expression analysis of down and upregulated Brachypodium distachyon genes. Expression profiling at 3-week seedling stage with 20% PEG osmotic pressure. The upregulation of BdDi19 was exceedingly higher than that of any other upregulated gene observed. Standard deviations were calculated from the mean values of three independent samples.

In order to have a broad idea of the plausibility of utilizing this artificial setup for genetic stress analysis several different genes were selected, 2 genes which are well established as upregulated, one was a predicted gene not experimentally validated, and 2 genes that were downregulated, as presented in Figure 8. No clear trend was observed at short-term osmotic stress over a period of 2 h. Since the expression pattern obtained for each gene was not definitive, we decided to proceed with greater osmotic stress over an extended period. 20% PEG was applied over a length of 24 h. The data obtained were in line with previous expression profiles of the selected genes. 

With more osmotic stress 20% PEG and longer stress duration (4, 6, 8, 12, 24 h) the expression profile of upregulated and downregulated genes under water stress was like that in previous data. BdNAC54 and BdNAC92 were downregulated as has been previously reported [26]. BdLEA5 is a gene upregulated in developing stages in plants, so its high expression was expected. DREB is a well-established drought-responsive gene [48,49] and was upregulated at 4 h and then displayed a 1.5-fold decrease at 6 h and remained more or less the same until 24 h. The most interesting observation was of the BdDi19 gene which is reported here the first time in Brachypodium distachyon. Its expression increased 120-fold after 24 h of osmotic stress as seen in Figure 8. Dehydration-Induced 19 protein is a characterized protein in Oryza sativa having conserved domains in most grass species including Aegilops tauschii, Hordeum vulgare, Setaria italica among many other Poaceae. This is the first time it has been experimentally verified to respond to drought in any plant species.

## 3. Discussion

Growth, directionality and compatibility were observed for Brachypodium seeds on all three PDMS punched molds, and the results were in line with the previous reports conducted with Nicotiana and Arabidopsis [12,19,50]. After several experiments, we concluded that after 4 days of vernalization and 2 DAG seedling stage, the seedling had to be inserted in the correct orientation in the chip to make it grow along the length of the narrow 1 mm channel. Growth was observed with the root penetrating the length of the microchannel with a slight curvature and bending.

A study on young wheat seedlings shows cell wall expansion in the maturation zone upon a low water potential around the roots and the authors suggest the accumulation of some solutes within the elongation and maturation zones in order to maintain the turgor pressure, resulting in an increase in the root diameter [46]. Although not seen in maturation zone cells, but a similar swelling behaviour of cells at the root apical meristem zone upon treatment with 5% PEG was previously reported for Brachypodium as well as wheat, rice, soybean, and maize [47], suggesting a collective response by root tissues of different plants to surmount the osmotic stress. The observation of Casparian strips in the endodermis is in line with results on waterlogging in Brachypodium since both stagnant water and dehydration with PEG are osmotic pressures on the cells [23].

Our results show the ease of visualization and utilization of neutral red as a promising dye for monocot PEG-mediated stress without tissue processing. Since Neutral red stains casparian tubes it is an excellent vital stain to be used in monocots for in situ analysis without processing the tissue or sample preparation. 

The Di19 protein was first reported in Oryza sativa as OsDi19-1 through RNA-Seq data. In Triticum aestivum, it was known to have a C terminal domain. At the N terminus is a zinc finger zf-Di19. It was reported to be involved in environmental stress, i.e., cold, drought, osmotic stress, and salinity. The protein was induced by high levels of abscisic acid and ethylene [51]. Though spatiotemporal expression analysis the stem root and leaf overall showed an increase in expression of OsDi19 as compared to all other tissues [52]. Under flooding and osmotic pressure, the expression also increased compared to other stress conditions such as salinity, cold, dryness, cadmium and hormones [53]. BdDi19 is thus a little-known gene which has a profound expression during short-term osmotic stress in young seedlings. Further studies on this gene and protein in Poaceae could be useful in understanding dehydration and osmotic stress in relation to development.

This is the first study to incorporate neutral red for Brachypodium morphological analysis; this is also the first study to analyse PEG-mediated stress with neutral red dye. Moreover, this is the first study to experimentally validate Dehydration Induced 19 protein and analyse the expression levels of BdDi19 under drought stress.

We propose that a follow-up study on Brachypodium seedlings in automated microfluidics or bioMEMS devices can be built upon our observations. Organ growth dynamics, root elasticity, microfluidic flow analysis and root hair dynamics under real time are a few of several areas which are desirable to be undertaken to unravel yet unknown physical patterns of growth and growth cessation and adaptation in favourable and stresses conditions.

## 4. Materials and Methods

### 4.1. Device Fabrication

Rectangular PDMS pieces with a scale of 65 × 20 × 10 mm single, double and triple punched with 5 mm diameter punchers were initial seed growing reservoirs at different volumes to check biocompatibility. Acetone cleaned glass slides, and PDMS pieces were plasma treated and bonded to get the final devices, which were used to test the compatibility of Brachypodium seeds with PDMS. A mold for the plant chip was designed with SOLIDWORKS Software, reproduced onto ABS 3D material, and 3D printed. The mold dimensions were 10 mm height, 9.5 mm channel length, 1 mm outlet diameter, and each seed channel 4 mm in diameter. The channel height was fixed at 1 mm to ensure the growth of the root to remain in one plane and not be out of focus in the Z-axis under microscopy as was earlier observed for 2 mm channel. For the construction of the device, PDMS and curing agent were mixed in 10:1 ratio and poured into the mold in a 100-mm diameter Petri dish, degassed in a desecrator, and cured at 75 °C for 60 min in an oven. The PDMS pieces were cut and gently peeled off from the mold on the Petri dish. The constructed device was submerged in Murashige and Skoog media overnight to ensure the hardening of the device. 0.17 mm coverslips and the PDMS pieces were plasma treated and bonded to get the final devices. Coverslips were used instead of the glass slides to facilitate fluorescent imaging. This setup was fixed with an adhesive to the Petri plate cover. Each channel was filled with MS media.

### 4.2. Preparation of Seeds and Measurement of Growth

Brachypodium wild-type seed line Bd21-3 was used in this study. The seeds were dehusked then soaked in water for 10 min. They were sterilised for 1 min with 70% ethanol in a sterile Petri dish. The ethanol was drained, and the seeds were rinsed with sterile deionized water. 20 mL of 1.3% NaOCl solution was poured into the Petri dish and rotated for 5 min. The seeds were then rinsed thrice with sterile deionized water. Ten seeds were placed in between two layers of sterile filter papers soaked in sterile water. It was observed that incubation at 4 °C for 7 days synchronised germination and promoted rapid growth as compared to 2d or 4d vernalization.

Media prepared was Murashige and Skoog 4.43 g, MES Monohydrate 0.5 g, Sucrose 30 g, and BAP 2.5 mg/L. After germination, the seeds were transferred to agar media and allowed to grow for 48 h at 22 °C with a 16 h photoperiod and high relative humidity at 57%. Finally, the seedlings were transferred to the device.

Epson perfection v700 photo scanner was used to visualise the full length of the seedlings grown in the microfluidic device and standard agar environment. WinRHIZO software (Regent Instruments, Quebec, Canada) was used to analyse the shoot and the root scan images.

For each channel, a seedling was grown in conventional Petri plates with Murashige and Skoog solid media in closed and sterile conditions. The channel was designed to restrain the root growth to a horizontally narrow path (1 mm diameter) which was optically transparent (0.17 mm glass coverslips). 4-days of vernalization and two days are post-germination synchronously growing Brachypodium seedlings were inserted into the wells vertically at around 75–55° angle, with the scutellum facing slightly upwards and radicula facing downward to allow growth imaging of the roots in the narrow horizontal channels. The anterior end was immersed in the well, and the posterior end was entirely out of the well, with the emerging leaf facing outwards. This provided gas exchange and illumination for the leaves. The potential of a plant-on-a-chip setup for Brachypodium seeds was shown in Figure S2.

### 4.3. Osmotic Stress Application

Osmotic stress was applied with 20% PEG 6000 that was poured into the MS agar media. 270 uL of the prepared stress media was placed in the 3-punch microchannel. The seedlings were initially grown in the microfluidic device for three weeks when the plant reached a three-leaf stage; then they were transferred into the 3-punch microchannel device filled with the stress media to ensure the roots were fully immersed in the PEG-MS.

For 6 h and 24 h osmotic stress analyses, the seedlings were first stained with neutral red for 20 min and then transferred to the preliminary microchannel device containing 20% PEG-MS and visualized under a fluorescence microscope.

### 4.4. Imaging Setups

The seedlings were selected 2 days after the germination for microscopy studies. For standard visualisation of the control samples and the samples under the osmotic stress, the device setup for top imaging was used. MS media with PEG-supplement was used for samples under the osmotic stress. The top imaging was performed using Nikon and Olympus stereo microscopes from Japan.

For bottom imaging of the samples with fluorescent, a stock solution of 4 µM neutral stain was prepared with 0.2X MS medium supplemented with 20 mM potassium phosphate buffer at 8.0 pH, according to the procedure reported earlier [43]. The samples with and without abiotic stress were stained for 15–20 min following the removal of PEG-supplemented MS media. The staining procedure made the cells slightly more visible and enabled fluorescent visualisation of the seedlings. The cross-section samples were prepared according to the protocol described online by Schiefelbein Lab [54]. Fluorescence imaging was performed with an Axio Vert.A1 inverted microscope by Carl Zeiss (Germany), using the microfluidic device with the bottom imaging setup.

The confocal microscopy was performed with Carl Zeiss LSM 710, Germany and images recorded with Zen software (Carl Zeiss Microscopy GmbH, Jena, Germany). A single channel was used for visualisation with neutral red. The images were taken in 20X objective lens. Three-week seedlings were selected which were already pre-stained with neutral red at the 2-day seedling stage (2 DAG-days after germination) (stained as mentioned previously in the article). These seedlings were given drought stress for 6 h in Murashige and Skoog media with 20% polyethylene glycol 6000. These were then embedded in agarose (as described for the fluorescent microscope staining) to enable section slicing as thin as possible. Cut sections ~0.5–0.9 mm were achieved. The maturation zone of the plant was selected. Transverse sections were removed from the agarose molds and placed separately on acetone-ethanol cleansed coverslips and glass slides. The coverslips were sealed securely with clear nail polish. 

### 4.5. RNA Isolation, DNase Treatment and qRT-PCR

RNA isolation was done from the whole plantlets with a Zymo research kit MiniPrep RNA isolation kit. Briefly, the tissue was homogenized in trizol reagent and transferred to column tubes. Flow through was DNase treated in the column, followed by prep buffer and wash buffers with intermittent centrifugations. The final eluted RNA was 20 uL. The concentration of RNA was verified by nanodrop spectrometer. A bleaching gel was used to analyse the integrity of the RNA. A 1% bleach gel was prepared in 1X TBE. 10X RNA loading buffer was used, and a low molecular weight RNA ladder was used.

From the plant genome database (http://www.plantgdb.org/prj/GenomeBrowser/) the Brachypodium genome was used to search for upregulated genes in drought. drought responsive family protein 19 (now renamed Dehydration Induced19) DI19, Late Embryogenesis abundant protein Lea5, sequences were downloaded, and primers designed. Primers for DREB2A were taken from Feng et al. 2015. Two downregulated genes BdNAC054 and BdNAC092 were selected, and primer sequences were taken from You et al. [26]. Ubiquitin BdUBC18 was taken as internal Control, and the primers used for it were also from You et al. [26]. Primer sequences were also presented in Table S1.

After confirmation of the integrity of the RNA samples, cDNA synthesis was performed with RevertAid First Strand cDNA Synthesis kit (Thermo Fisher Scientific, Waltham MA, USA) following the manufacturer’s instructions. Quantitative reverse transcription polymerase chain reaction (qRT-PCR) was performed by Light Cycler 480 II Instrument (Roche Diagnostics GmbH, Mannheim, Germany) using Perfecta SYBR Green SuperMix from Quanta Biosciences, Gaithersburg, MD, USA. Amplification was performed in a total reaction volume of 10 µL containing 5 µL of 1X SYBR Green Super Mix, 300 nM of each primer, and 100 ng of the template cDNA. The PCR thermal cycling parameters were set at 95 °C for 10 min to activate the SYBR green followed by 40 cycles of 95 °C for 15 s and 60 °C for 1 min. For each sample, three technical replicates were made. Plant samples without stress treatment (0 h) was used for normalization and fold change calculation. The ΔΔCt Pfaffel method was used to analyse the relative gene expression of the qPCR results.

## Figures and Tables

**Table 1:** Microfluidic devices developed so far for the in-depth optical analysis of plant samples.

Species Name	Species	Organ Studied	Device Type	Physical Parameters	Ref.
*Camellia japonica*	Dicot	Pollen tube	Lab-on-a-chip (LOC) technology	Influence of electric fields and conductivity	[15]
	Dicot, fungus	Pollen grains, root hairs or fungal spores	Tip Chip (serially arranged microchannels)	Experimentation and phenotyping of chemical gradients, microstructural features, integrated biosensors or directional triggers within the modular microchannels	[16]
	Dicot	Pollen grains	Microchannels and inlets/outlets	Protuberance growth of single plant cells in a micro- vitro environment	[36]
	Dicot	Pollen grains	Tip Chip	penetrative forces generated in pollen tubes	[37]
	Dicot	Pollen tube	Laminar flow based microfluidic device	Ca^2+,^ pectin methylesterase (PME) application for quantitative assessment of chemoattraction	[38]
	Dicot	Pollen tube	Device with a knot-shaped microchannel microfluidic	Trapping probability and uniformity of fluid flow conditions	[17]
	Dicot	Pollen tube	Trapping microfluidic device	Primary and secondary peak frequencies in oscillatory growth dynamics	[14]
	Dicot	Pollen tube	Bending-Lab-On-a-Chip (BLOC)	Flexural rigidity of the pollen tube and Young’s modulus of the cell wall	[39]
*Arabidopsis thaliana*	Dicot	Plant body/Root	Microfluidic chip platform Root Chip	Monitoring time-resolved growth and cytosolic sugar levels at a subcellular resolution	[8]
	Dicot	Embryo	PDMS micropillar array	Live-Cell Imaging and Optical Manipulation	[9]
	Dicot	Root/Plants	Root Array	Imaged by confocal microscopy	[6]
	Dicot	Root	RootChip16	Identification of defined [Ca^2+^] cyt oscillations, Forster resonance energy transfer (FRET)	[10]
	Dicot	Plant body- pathogen interaction	Plant Chip: vertical and transparent microfluidic for high-throughput phenotyping	Quantitative monitoring of plant phenotypes	[11]
	Dicot	Live Root	Plant on-chip platform	Stimuli and Phytohormones 2,4-dichlorophenoxyacetic acid (2,4-D), and its inhibitorN-1-naphthylphthalamic acid (NPA)	[12]
	Dicot	Pollen-ovule	Mimicry of in vivo microenvironment of ovule fertilization	Chemoattraction	[7]
*Torenia fournieri*	Dicot	Pollen tube, ovules	T-shaped microchannel device, micro cage array	Pollen tube chemoattraction, long-term live imaging of ovules	[40]
	Dicot	Pollen tubes	T-shaped channel	Quantitate the effect of chemoattractants on directional pollen tube growth, UV-irradiation	[41]
	Dicot	Pollen Tube	Crossroad device	Net guidance response ratio (GRR)	[42]
*Tobacco Nicotiana tabacum*	Dicot	Mesophyll Protoplast	Microcolumn array	Microscopic real-time optimization and dynamics of protoplast growth including size change, organelle motion, and cell mass formation	[20]
*Phalaenopsis*	Dicot	Protoplasts	Convex-concave sieving array	Real-time collection and lysis of *Phalaenopsis* protoplasts	[21]

## Supplementary Materials

The following are available online at http://www.mdpi.com/2223-7747/8/1/14/s1, Figure S1: Testing Brachypodium seedlings for orientation, compatibility and growth, Figure S2: Potential of a plant-on-a-chip setup for Brachypodium seeds. Table S1: Primers used for qRT-PCR.

## References

[1] Frazier T.P., Sun G., Burklew C.E., Zhang B. (2011). Salt and drought stresses induce the aberrant expression of microRNA genes in tobacco. Mol. Biotechnol..

[2] Kumar R.R., Pathak H., Sharma S.K., Kala Y.K., Nirjal M.K., Singh G.P., Goswami S., Rai R.D. (2015). Novel and conserved heat-responsive microRNAs in wheat (*Triticum aestivum* L.). Funct. Integr. Genom..

[3] Verelst W., Bertolini E., De Bodt S., Vandepoele K., Demeulenaere M., Enrico Pè M., Inzé D. (2013). Molecular and Physiological Analysis of Growth-Limiting Drought Stress in Brachypodium distachyon Leaves. Mol. Plant.

[4] Elitaş M., Yüce M., Budak H. (2017). Microfabricated tools for quantitative plant biology. Analyst.

[5] Sanati Nezhad A. (2014). Microfluidic platforms for plant cells studies. Lab Chip.

[6] Busch W., Moore B.T., Martsberger B., Mace D.L., Twigg R.W., Jung J., Pruteanu-malinici I., Kennedy S.J., Gregory K., Nc D. (2012). A microfluidic device and computational platform for high throughput live imaging of gene expression. Nat. Methods.

[7] Yetisen A.K., Jiang L., Cooper J.R., Qin Y., Palanivelu R., Zohar Y. (2011). A microsystem-based assay for studying pollen tube guidance in plant reproduction. J. Micromech. Microeng..

[8] Grossmann G., Guo W.-J., Ehrhardt D.W., Frommer W.B., Sit R.V., Quake S.R., Meier M. (2011). The RootChip: An integrated microfluidic chip for plant science. Plant Cell.

[9] Gooh K., Ueda M., Aruga K., Park J., Arata H., Higashiyama T., Kurihara D. (2015). Live-Cell Imaging and Optical Manipulation of Arabidopsis Early Embryogenesis. Dev. Cell.

[10] Keinath N., Waadt R., Brugman R., Schroeder J.I., Grossmann G., Schumacher K., Krebs M. (2015). Live cell imaging with R-GECO1 sheds light on flg22- and chitin-induced transient [Ca^2+^]cyt patterns in Arabidopsis. Mol. Plant.

[11] Jiang H., Xu Z., Aluru M.R., Dong L. (2014). Plant chip for high-throughput phenotyping of Arabidopsis. Lab Chip.

[12] Meier M., Lucchetta E.M., Ismagilov R.F. (2010). Chemical stimulation of the Arabidopsis thaliana root using multi-laminar flow on a microfluidic chip. Lab Chip.

[13] Massalha H., Korenblum E., Malitsky S., Shapiro O.H., Aharoni A. (2017). Live imaging of root–bacteria interactions in a microfluidics setup. Proc. Natl. Acad. Sci. USA.

[14] Nezhad A.S., Packirisamy M., Bhat R., Geitmann A. (2013). In Vitro Study of Oscillatory Growth Dynamics of Camellia Pollen Tubes in Microfluidic Environment. IEEE Trans. Biomed. Eng..

[15] Agudelo C.G., Packirisamy M., Geitmann A. (2014). Assessing the Influence of Electric Cues and Conductivity on Pollen Tube Growth via Lab-On-A-Chip Technology. Biophys. J..

[16] Agudelo C.G., Sanati Nezhad A., Ghanbari M., Naghavi M., Packirisamy M., Geitmann A. (2013). TipChip: A modular, MEMS-based platform for experimentation and phenotyping of tip-growing cells. Plant J..

[17] Ghanbari M., Nezhad A.S., Agudelo C.G., Packirisamy M., Geitmann A. (2014). Microfluidic positioning of pollen grains in lab-on-a-chip for single cell analysis. J. Biosci. Bioeng..

[18] Iyer-Pascuzzi A.S., Symonova O., Mileyko Y., Hao Y., Belcher H., Harer J., Weitz J.S., Benfey P.N. (2010). Imaging and Analysis Platform for Automatic Phenotyping and Trait Ranking of Plant Root Systems. Plant Physiol..

[19] Ko J.-M., Ju J., Lee S., Cha H.-C. (2006). Tobacco protoplast culture in a polydimethylsiloxane-based microfluidic channel. Protoplasma.

[20] Wu H., Liu W., Tu Q., Song N., Li L., Wang J.J., Wang J.J. (2011). Culture and chemical-induced fusion of tobacco mesophyll protoplasts in a microfluidic device. Microfluidics Nanofluidics.

[21] Hung M.-S., Chang J.-H. (2012). Developing microfluidics for rapid protoplasts collection and lysis from plant leaf. Proc. Inst. Mech. Eng. Part N J. Nanoeng. Nanosyst..

[22] Bascom C.S., Wu S.-Z., Nelson K., Oakey J., Bezanilla M. (2016). Long-Term Growth of Moss in Microfluidic Devices Enables Subcellular Studies in Development. Plant Physiol..

[23] Shiono K., Yamada S. (2014). Waterlogging tolerance and capacity for oxygen transport in Brachypodium distachyon (Bd21). Plant Root.

[24] Hardtke C.S., Pacheco-Villalobos D., Vogel J.P. (2016). The Brachypodium distachyon Root System: A Tractable Model to Investigate Grass Roots. Genetics and Genomics of Brachypodium.

[25] Hong S.-Y., Seo P., Yang M.-S., Xiang F., Park C.-M. (2008). Exploring valid reference genes for gene expression studies in Brachypodium distachyon by real-time PCR. BMC Plant Biol..

[26] You J., Zhang L., Song B., Qi X., Chan Z. (2015). Systematic Analysis and Identification of Stress-Responsive Genes of the NAC Gene Family in Brachypodium distachyon. PLoS ONE.

[27] Gordon S.P., Priest H., Des Marais D.L., Schackwitz W., Figueroa M., Martin J., Bragg J.N., Tyler L., Lee C.-R., Bryant D. (2014). Genome diversity in Brachypodium distachyon: Deep sequencing of highly diverse inbred lines. Plant J..

[28] Vogel J.P., Garvin D.F., Mockler T.C., Schmutz J., Rokhsar D., Bevan M.W., Barry K., Lucas S., Harmon-Smith M., Lail K. (2010). Genome sequencing and analysis of the model grass Brachypodium distachyon. Nature.

[29] Budak H., Hernandez P., Schulman A. (2014). Analysis and Exploitation of Cereal Genomes with the Aid of Brachypodium. Genomics of Plant Genetic Resources.

[30] Brkljacic J., Grotewold E., Scholl R., Mockler T., Garvin D.F., Vain P., Brutnell T., Sibout R., Bevan M., Budak H. (2011). Brachypodium as a Model for the Grasses: Today and the Future. Plant Physiol..

[31] Girin T., David L.C., Chardin C., Sibout R., Krapp A., Ferrario-Méry S., Daniel-Vedele F. (2014). Brachypodium: A promising hub between model species and cereals. J. Exp. Bot..

[32] Filiz E., Ozdemir B.S., Budak F., Vogel J.P., Tuna M., Budak H. (2009). Molecular, morphological, and cytological analysis of diverse Brachypodium distachyon inbred lines. Genome.

[33] Oliveira E.J., Koehler A.D., Rocha D.I., Vieira L.M., Pinheiro M.V.M., de Matos E.M., da Cruz A.C.F., da Silva T.C.R., Tanaka F.A.O., Nogueira F.T.S. (2017). Morpho-histological, histochemical, and molecular evidences related to cellular reprogramming during somatic embryogenesis of the model grass Brachypodium distachyon. Protoplasma.

[34] Barrero J.M., Jacobsen J.V., Talbot M.J., White R.G., Swain S.M., Garvin D.F., Gubler F. (2012). Grain dormancy and light quality effects on germination in the model grass Brachypodium distachyon. New Phytol..

[35] Guillon F., Larré C., Petipas F., Berger A., Moussawi J., Rogniaux H., Santoni A., Saulnier L., Jamme F., Miquel M. (2012). A comprehensive overview of grain development in Brachypodium distachyon variety Bd21. J. Exp. Bot..

[36] Nezhad A.S., Ghanbari M., Agudelo C.G., Naghavi M., Packirisamy M., Bhat R.B., Geitmann A. (2014). Optimization of flow assisted entrapment of pollen grains in a microfluidic platform for tip growth analysis. Biomed. Microdevices.

[37] Nezhad A.S., Naghavi M., Packirisamy M., Bhat R., Geitmann A., Sanati Nezhad A., Naghavi M., Packirisamy M., Bhat R., Geitmann A. (2013). Quantification of cellular penetrative forces using lab-on-a-chip technology and finite element modeling. Proc. Natl. Acad. Sci. USA.

[38] Nezhad A.S., Packirisamy M., Geitmann A. (2014). Dynamic, high precision targeting of growth modulating agents is able to trigger pollen tube growth reorientation. Plant J..

[39] Nezhad A.S., Naghavi M., Packirisamy M., Bhat R., Geitmann A. (2013). Quantification of the Young’s modulus of the primary plant cell wall using Bending-Lab-On-Chip (BLOC). Lab Chip.

[40] Arata H., Higashiyama T. (2014). Poly(dimethylsiloxane)-based microdevices for studying plant reproduction. Biochem. Soc. Trans..

[41] Horade M., Kanaoka M.M., Kuzuya M., Higashiyama T., Kaji N. (2013). A microfluidic device for quantitative analysis of chemoattraction in plants. RSC Adv..

[42] Sato Y., Sugimoto N., Higashiyama T., Arata H. (2015). Quantification of pollen tube attraction in response to guidance by female gametophyte tissue using artificial microscale pathway. J. Biosci. Bioeng..

[43] Dubrovsky J.G., Guttenberger M., Saralegui A., Napsucialy-Mendivil S., Voigt B., Baluska F., Menzel D. (2006). Neutral red as a probe for confocal laser scanning microscopy studies of plant roots. Ann. Bot..

[44] Yazdanbakhsh N., Sulpice R., Graf A., Stitt M., Fisahn J. (2011). Circadian control of root elongation and C partitioning in Arabidopsis thaliana. Plant. Cell Environ..

[45] Paez-Garcia A., Motes C., Scheible W.-R., Chen R., Blancaflor E., Monteros M. (2015). Root Traits and Phenotyping Strategies for Plant Improvement. Plants.

[46] Akmal M., Hirasawa T. (2004). Growth responses of seminal roots of wheat seedlings to a reduction in the water potential of vermiculite. Plant Soil.

[47] Ji H., Liu L., Li K., Xie Q., Wang Z., Zhao X., Li X. (2014). PEG-mediated osmotic stress induces premature differentiation of the root apical meristem and outgrowth of lateral roots in wheat. J. Exp. Bot..

[48] Hackenberg M., Gustafson P., Langridge P., Shi B.J. (2015). Differential expression of microRNAs and other small RNAs in barley between water and drought conditions. Plant Biotechnol. J..

[49] Jiang Y., Wang X., Yu X., Zhao X., Luo N., Pei Z., Liu H., Garvin D.F. (2017). Quantitative Trait Loci Associated with Drought Tolerance in Brachypodium distachyon. Front. Plant Sci..

[50] Lei R., Qiao W., Hu F., Jiang H., Zhu S. (2015). A simple and effective method to encapsulate tobacco mesophyll protoplasts to maintain cell viability. MethodsX.

[51] Li S., Xu C., Yang Y., Xia G. (2010). Functional analysis of TaDi19A, a salt-responsive gene in wheat. Plant. Cell Environ..

[52] Rice Expression Data Spatio-Temporal Gene Expression of Various Tissues/Organs throughout Entire growth in the Field. http://ricexpro.dna.affrc.go.jp/GGEP/graph-view.php?featurenum=12093#tabs-2.

[53] Kawahara Y., Oono Y., Wakimoto H., Ogata J., Kanamori H., Sasaki H., Mori S., Matsumoto T., Itoh T. (2016). TENOR: Database for Comprehensive mRNA-Seq Experiments in Rice. Plant Cell Physiol..

[54] Schiefelbein Lab Rapid Preparation of Transverse Sections of Plant Roots|Schiefelbein Lab. http://sites.lsa.umich.edu/schiefelbein-lab/rapid-preparation-of-transverse-sections-of-plant-roots/.

