# Supplementary material for: THROUGH THE LOOKING GLASS: Real-Time Imaging in Brachypodium Roots and Osmotic Stress Analysis
Source: Plants (Basel). 2019 Jan 8;8(1):14. doi: 10.3390/plants8010014 (PMC6358813; doi:10.3390/plants8010014)
Supplement: Supplementary file 1 [file plants-08-00014-s001.zip › plants-399994-SI/plants-399994-sup-revise2.docx]

**THROUGH THE LOOKING GLASS: Real-time imaging in Brachypodium roots and osmotic stress analysis**

**Zaeema Khan ^1^, Hande Karamahmutoğlu ^2^, Meltem Elitaş ^2^, Meral Yüce ^3^ and Hikmet Budak ^4^***

^1^ Molecular Biology, Genetics and Bioengineering Program, Faculty of Engineering and Natural Sciences, Sabanci University, Istanbul, 34956, Turkey

^2^ Mechatronics Program, Faculty of Engineering and Natural Sciences, Sabanci University, Istanbul, 34956, Turkey

^3^ Sabanci University SUNUM Nanotechnology Research and Application Centre, 34956, Istanbul, Turkey

^4^ Cereal Genomics Lab, Department of Plant Sciences and Plant Pathology, Montana State University, Bozeman, 59717, USA

* Corresponding author e-mail: hikmet.budak@montana.edu; Tel: 406-994-6717


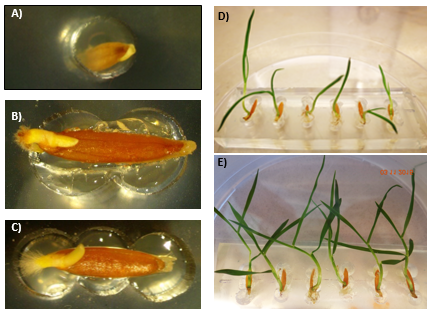


**Figure S1. Testing Brachypodium seedlings for orientation, compatibility and growth.** The growth of monocot seedlings from Brachypodium distachyon in singe **(A)**, double and triple **(C)** punched PDMS channel, growth of six samples in parallel after 7 days in the triple punched PDMS channel **(D)**, growth of six samples in parallel after 21 days in the triple-punched PDMS channel **(E)**.


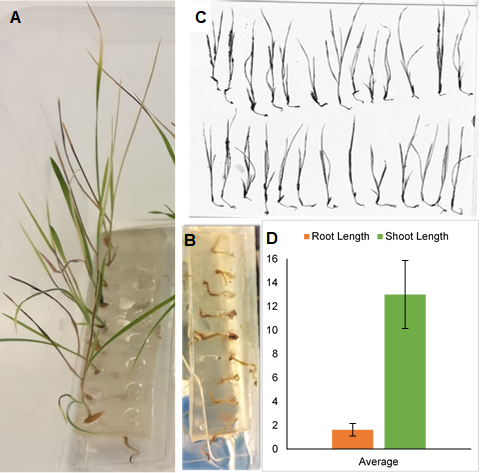


**Figure S2.** **Potential of a plant-on-a-chip setup for Brachypodium seeds.** **A)** Growth at >3 weeks, **B)** showing the root growth in a single plane but hindered due to the channel. **C)** The maximum growth obtained after 3 weeks showing potential for a root array arrangement and maintenance for a month. D) The average growth of the plants roots and shoots obtained from the array.

In **Figure S2** we analyzed the maximum extent to which we could maintain the Brachypodium plantlets inside the artificial setup environment. The graph shows the average growth of the plant's roots and shoots obtained from the array obtained from 26 seedlings after 1-month growth in PDMS, however extensive browning was observed at this stage thus we propose to analyze healthy green plantlets the experiment should not last more than 3 weeks. The average height of the leaf was recorded as 13 cm, average root length 1.63 cm, maximum shoot length of 22.5 cm and root length of 2.6 cm was obtained after 4 weeks growth, which we propose as the maximum period to maintain the Brachypodium seedlings in the device. Our purpose was to approach the maximum limit until the leaf senescence would start to manifest. The apparatus gives the potential of a plant-on-a-chip device. Furthermore, enough downstream applications such as stress application and RNA isolation for gene expression analysis.

**Table S1 Primers used for qRT-PCR**

| BdNAC054 | F: 5’- CAATTCAAAACTGGAGCTGCC -3’  R: 5’- CATCTACTTCACTACCAGCACG -3’ | [1] |
| --- | --- | --- |
| BdNAC092 | F: 5’- GATGGAAGACACGATAGGGAC -3’  R: 5’- TGTTGGAGAAGCAGGTCAC -3’ |  |
| BdUBC18 | F: 5’- ACCGCTATGTTAGGAACTGC  R: 5’- TGACTGGCTAATATGGTGTGG -3’ |  |
| DREB2A | F: 5’- ACCTCAGCACCTTCATTGCT -3’  R: 5’- TTGCCCTCCTGTTGGAATAC -3’ | [2] |
| BdLEA5 | F: 5’- CTCTCGCTCTCTCCGGTTC -3’  R: 5’- GTAGCTTCTGGTGGACGGG -3’ | --- |
| DRFP19 | F: 5’- AGCCGATCCTCTCCTGTCAT -3’  R: 5’- TTCTTCGCTGGTGAGGGATG -3’ | --- |

**References**

1. You, J.; Zhang, L.; Song, B.; Qi, X.; Chan, Z. Systematic Analysis and Identification of Stress-Responsive Genes of the NAC Gene Family in Brachypodium distachyon. *PLoS One* **2015**, *10*, e0122027, doi:10.1371/journal.pone.0122027.

2. Feng, Y.; Yin, Y.; Fei, S. Down-regulation of BdBRI1, a putative brassinosteroid receptor gene produces a dwarf phenotype with enhanced drought tolerance in Brachypodium distachyon. *Plant Sci.* **2015**, *234*, 163–173, doi:10.1016/j.plantsci.2015.02.015.
