# Supplementary material for: THROUGH THE LOOKING GLASS: Real-Time Imaging in Brachypodium Roots and Osmotic Stress Analysis
Source: Plants (Basel). 2019 Jan 8;8(1):14. doi: 10.3390/plants8010014 (PMC6358813; doi:10.3390/plants8010014)
Supplement: Supplementary file 1 [file plants-08-00014-s001.zip › plants-399994-SI/Video S1.pptx]

## Slide 1
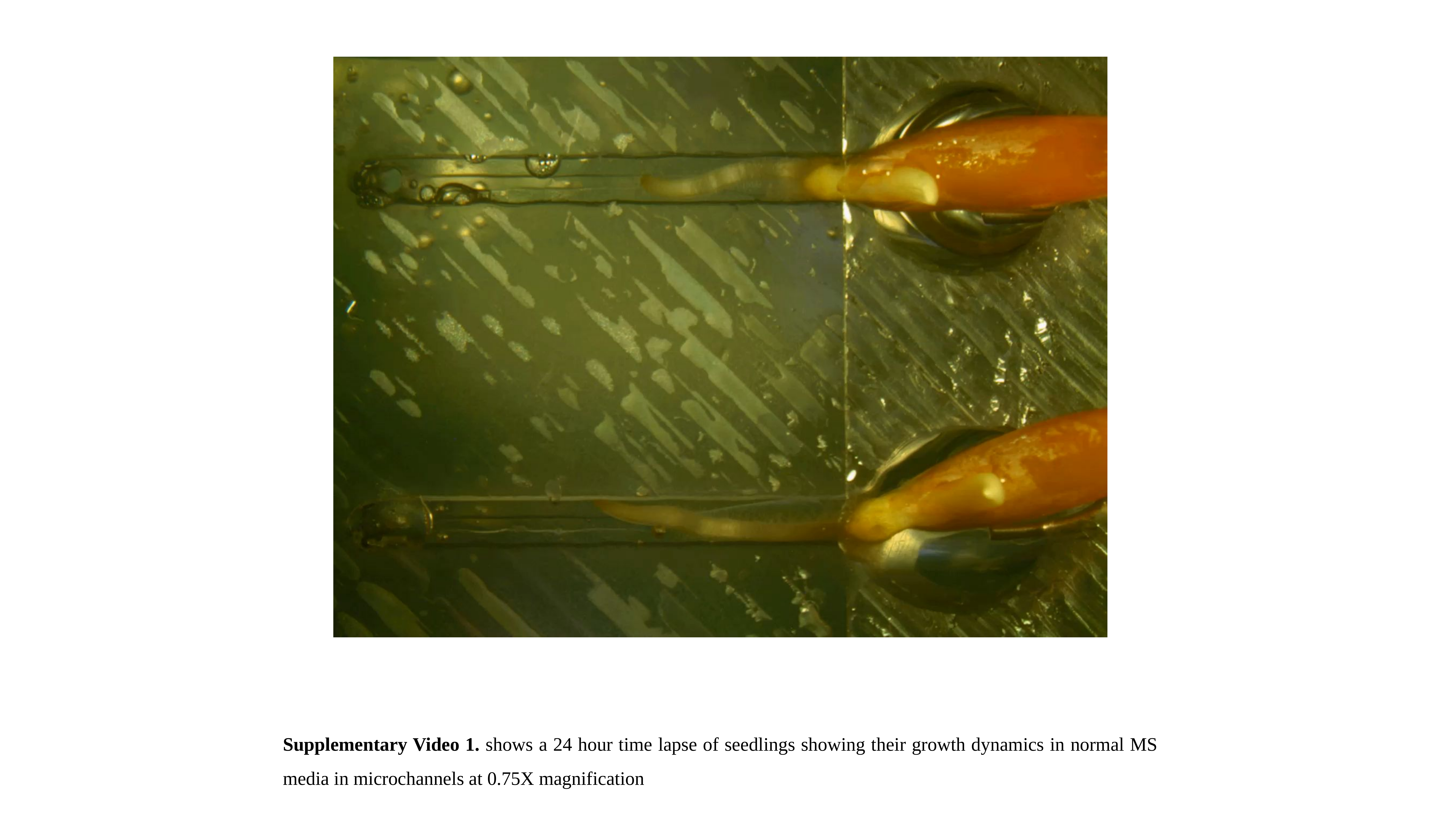

Supplementary Video 1. shows a 24 hour time lapse of seedlings showing their growth dynamics in normal MS media in microchannels at 0.75X magnification
